# 1-[(*E*)-2-(2-Hy­droxy-5-methyl­phen­yl)diazen-2-ium-1-yl]naphthalen-2-olate

**DOI:** 10.1107/S160053681302014X

**Published:** 2013-07-27

**Authors:** Souheyla Chetioui, Issam Boudraa, Sofiane Bouacida, Abdelkader Bouchoul, Salah Eddine Bouaoud

**Affiliations:** a Unité de Recherche de Chimie de l’Environnement et Moléculaire Structurale, (CHEMS), Faculté des Sciences Exactes, Département de Chimie, Université Constantine 1, 25000 Constantine, Algeria

## Abstract

The title zwitterion, C_17_H_14_N_2_O_2_, crystallizes with two independent mol­ecules in the asymmetric unit, both of which are approximately planar, the dihedral angles between the benzene ring and the naphthalene ring system being 4.39 (12)° in one mol­ecule and 5.83 (12)° in the other, and show an *E* conformation with respect to the azo double bond. An intra­molecular N—H⋯O hydrogen bond in each molecule helps to establish their near planar conformation. In the crystal, mol­ecules are linked through O—H⋯O hydrogen bonds into infinite chains running along the *a-*axis direction. In addition, the chains are stacked along the *b* axis *via* π–π inter­actions between the benzene and the naphthalene rings of adjacent mol­ecules, the centroid–centroid distances being 3.722 (3) and 3.823 (4) Å.

## Related literature

For general background to the use of azo compounds as dyes, pigments and advanced materials, see: Lee *et al.* (2004 ▶). For details of azo pigments, see: Herbst & Hunger (2004 ▶). For related structures of hydrazone derivatives, see: Olivieri *et al.* (1989 ▶); Oakes (2002 ▶). For bond-length data, see: Yazıcı *et al.* (2010 ▶); Karadayı *et al.* (2006 ▶). Many azo compounds have been synthesized by diazo­tization and diazo coupling reactions, see: Wang *et al.* (2003 ▶).
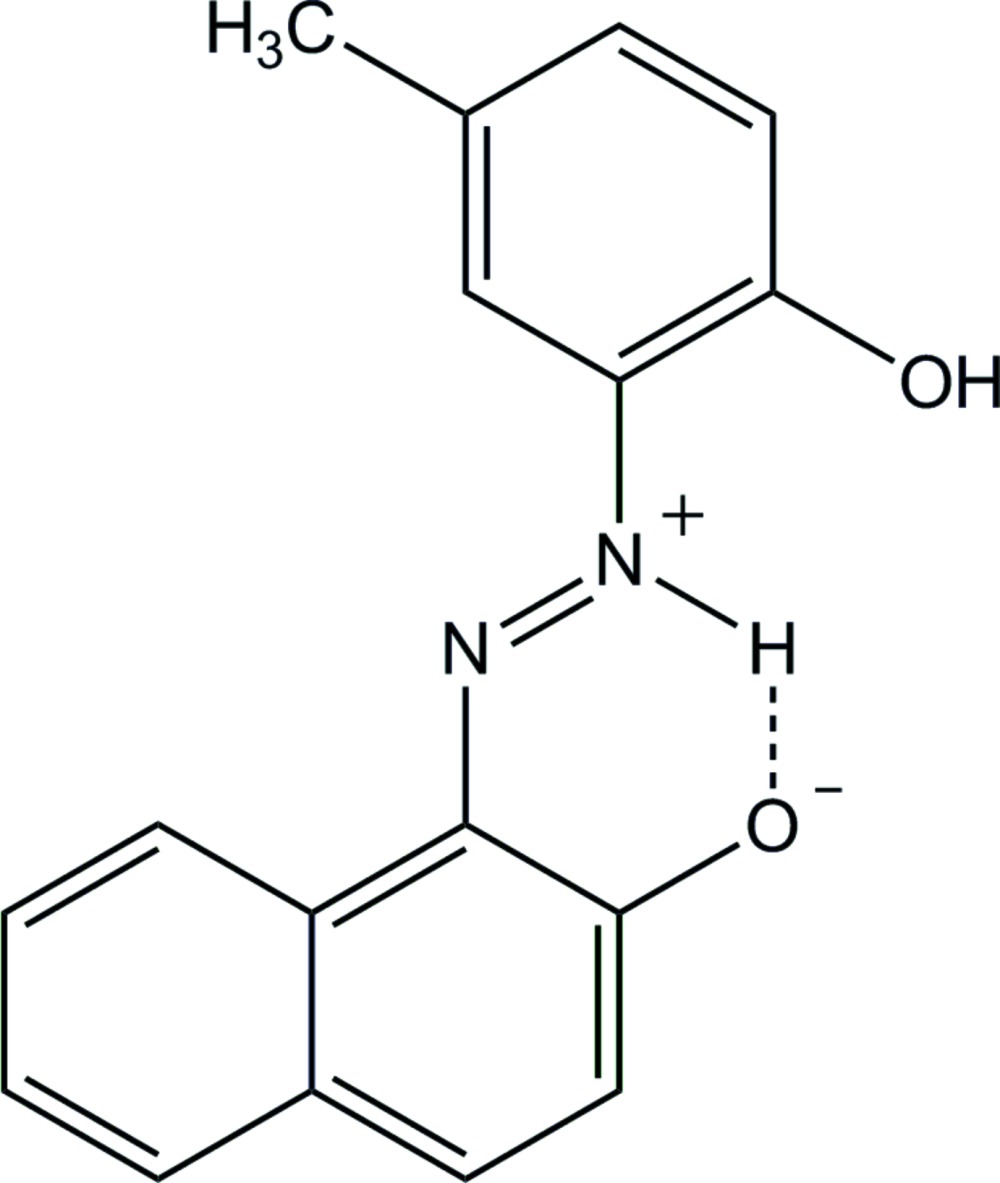


## Experimental

### 

#### Crystal data

C_17_H_14_N_2_O_2_
*M* *_r_* = 278.31
Monoclinic, 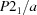

*a* = 14.541 (5) Å
*b* = 6.052 (5) Å
*c* = 32.633 (5) Å
β = 101.871 (5)°
*V* = 2810 (3) Å^3^
*Z* = 8
Mo *K*α radiation
μ = 0.09 mm^−1^
*T* = 150 K
0.03 × 0.02 × 0.02 mm

#### Data collection

Bruker APEXII diffractometer
Absorption correction: multi-scan (*SADABS*; Sheldrick, 2002 ▶) *T*_min_ = 0.853, *T*_max_ = 0.995
20440 measured reflections
6447 independent reflections
3301 reflections with *I* > 2σ(*I*)
*R*_int_ = 0.078

#### Refinement

*R*[*F* ^2^ > 2σ(*F* ^2^)] = 0.068
*wR*(*F* ^2^) = 0.202
*S* = 1.05
6447 reflections
386 parameters
2 restraints
All H-atom parameters refined
Δρ_max_ = 0.44 e Å^−3^
Δρ_min_ = −0.40 e Å^−3^

### 

Data collection: *APEX2* (Bruker, 2006 ▶); cell refinement: *SAINT* (Bruker, 2006 ▶); data reduction: *SAINT*; program(s) used to solve structure: *SIR97* (Altomare *et al.*, 1999 ▶); program(s) used to refine structure: *SHELXL97* (Sheldrick, 2008 ▶); molecular graphics: *ORTEP-3 for Windows* (Farrugia, 2012 ▶); software used to prepare material for publication: *WinGX* (Farrugia, 2012 ▶).

## Supplementary Material

Crystal structure: contains datablock(s) Chetioui-data, I. DOI: 10.1107/S160053681302014X/lr2112sup1.cif

Structure factors: contains datablock(s) I. DOI: 10.1107/S160053681302014X/lr2112Isup2.hkl

Supplementary material file. DOI: 10.1107/S160053681302014X/lr2112Isup3.cml

Additional supplementary materials:  crystallographic information; 3D view; checkCIF report

## Figures and Tables

**Table 1:** Hydrogen-bond geometry (Å, °)

*D*—H⋯*A*	*D*—H	H⋯*A*	*D*⋯*A*	*D*—H⋯*A*
N1—H1⋯O1	0.88 (2)	1.82 (3)	2.536 (4)	138 (2)
O2—H2⋯O6	0.82	1.85	2.631 (3)	159
O5—H5⋯O1^i^	0.82	1.81	2.622 (3)	168
N6—H6⋯O6	0.88 (2)	1.82 (3)	2.546 (4)	138 (2)

Symmetry code: (i) 
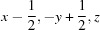
.

**Fig. 1.**
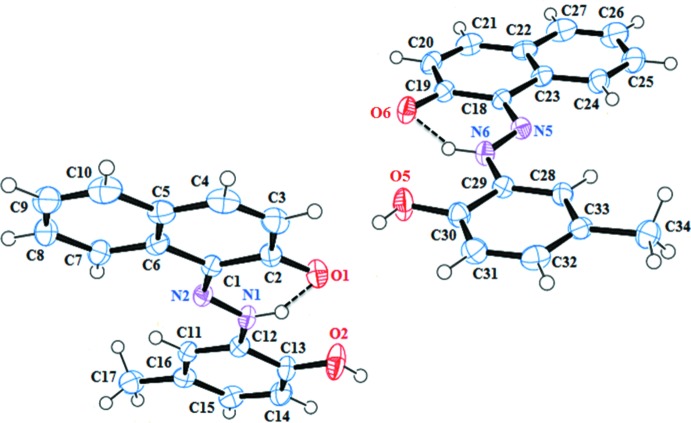
The molecular structure of (I), with 50% probability displacement ellipsoids for non-H atoms.The dashed lines indicate N—H···O hydrogen bond.

**Fig. 2.**
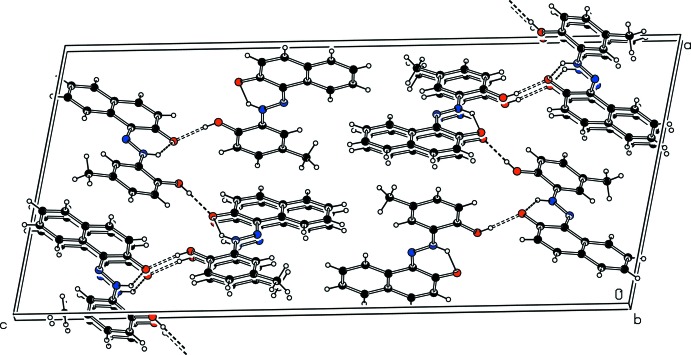
A view of the crystal packing showing the infinite one-dimensional chains.

## supplementary crystallographic information

### Comment

Azo compounds are very important in the fields of dyes, pigments and advanced materials (Lee *et al.*, 2004). Azo dyes are synthetic colours that contain an azo group, as part of the structure. We are involved in the color generation mechanism of azo pigments typically characterized by the chromophore of the azo group (–N=N–). However, some types of azo pigments are also known to possess the hydrazone structure (=N–NH–), often leading to the formation of intramolecular hydrogen bonds (Herbst & Hunger 2004). The azo– hydrazone tautomerism in azo dyes has been known for more than a hundred years and is directly connected with the presence of at least one protic donor group in conjugation to the azo bridge (*i.e.* 2-naphthol) (Olivieri *et al.*., 1989). In particular, azo dyes that contain a naphtholic hydroxy group conjugated with the azo linkage exist in aqueous solution as an equilibrium mixture of two chemically distinct tautomers, the azo or hydrazone forms (Oakes, 2002).It is suggested that in a real azo compound the N=N double bond should have a length of 1.20–1.28 Å and the bond length of N–N single bonds, as in hydrazone tautomers, should be more than 1.4 Å. In the title compound, N–N bond lengths are 1.385 Å for N1–N2 and 1.305 Å for N5–N6, between the suggested N=N double bond and N–N single bond lengths.In the molecule, all bond lengths are in good agreement with those reported for other azo compounds (Yazıcı *et al.*, 2010; Karadayı *et al.*, 2006).

The molecular structure of the title compound is shown in Figure 1.There are two independent molecules in the asymmetric unit, each consists of a benzene ring linked to the first nitrogen atom of the N = N chromophore and two aromatic rings of the core 2-naphthol, with a *trans* configuration with respect to the azo double bond. The dihedral angles between the benzene ring and naphthalene ring system being 4.39 (12)° in one molecule and 5.83 (12)° in the other. Intramolecular N—H···O hydrogen bonds stabilize the planar geommetry in each of the independent molecules. In the crystal, the molecules are linked through O—H···O into infinite one-dimensional chains running along the *a* axis, Figure 2. In addition, the chains are stacked along the *b* axis *via*π-π interactions between the benzene and the naphthalene rings of adjacent molecules, the centroid-centroid distances being 3.722 (3) and 3.823 (4) Å.

### Experimental

The title compound (*E*)-1-((2-hydroxy-5-methylphenyl)diazenyl)naphthalen-2-ol was prepared following the classical method of synthesis of other aromatic azo-compounds (Wang *et al.*, 2003). Diazotization of 2-amino-4-methylphenol followed by a coupling reaction with 2-naphthol. This gives a red powder which was recrystallized from ethanol leading to crystals in the form of red prisms.

### Refinement

All non-hydrogen atoms were refined with anisotropic atomic displacement parameters. All H atoms, attached to carbo(n atoms have been placed in geometrically idealized positions and refined as riding, with C—H = 0.93 (aromatic), 0.96 Å (methyl), and *U*_iso_(H) = 1.2 *U*_eq_(C) or 1.5 *U*_eq_(methyl C). Hydroxyl H atoms were introduced in calculated positions and treated as riding on their parent atoms with O—H = 0.82 Å (hydroxyl) and *U*_iso_(H) = 1.5 *U*_eq_(O). The remaining H atoms of amino-group were located in a difference Fourier map and refined freely with N—H= 0.88 Å and *U*_iso_(H) = 1.2 *U*_eq_(N).

### Crystal data

**Table:**

C_17_H_14_N_2_O_2_	*F*(000) = 1168
*M**_r_* = 278.31	*D*_x_ = 1.316 Mg m^−^^3^
Monoclinic, *P*2_1_/*a*	Mo *K*α radiation, λ = 0.71073 Å
Hall symbol: -P 2yab	Cell parameters from 2052 reflections
*a* = 14.541 (5) Å	θ = 3.1–28.6°
*b* = 6.052 (5) Å	µ = 0.09 mm^−^^1^
*c* = 32.633 (5) Å	*T* = 150 K
β = 101.871 (5)°	Prism, red
*V* = 2810 (3) Å^3^	0.03 × 0.02 × 0.02 mm
*Z* = 8	

### Data collection

**Table:**

Bruker APEXII diffractometer	6447 independent reflections
Radiation source: fine-focus sealed tube	3301 reflections with *I* > 2σ(*I*)
Graphite monochromator	*R*_int_ = 0.078
CCD rotation images, thin slices ω scans	θ_max_ = 27.6°, θ_min_ = 2.6°
Absorption correction: multi-scan (*SADABS*; Sheldrick, 2002)	*h* = −15→18
*T*_min_ = 0.853, *T*_max_ = 0.995	*k* = −7→7
20440 measured reflections	*l* = −42→42

### Refinement

**Table:**

Refinement on *F*^2^	Secondary atom site location: difference Fourier map
Least-squares matrix: full	Hydrogen site location: inferred from neighbouring sites
*R*[*F*^2^ > 2σ(*F*^2^)] = 0.068	All H-atom parameters refined
*wR*(*F*^2^) = 0.202	*w* = 1/[σ^2^(*F*_o_^2^) + (0.0877*P*)^2^] where *P* = (*F*_o_^2^ + 2*F*_c_^2^)/3
*S* = 1.05	(Δ/σ)_max_ < 0.001
6447 reflections	Δρ_max_ = 0.44 e Å^−^^3^
386 parameters	Δρ_min_ = −0.40 e Å^−^^3^
2 restraints	Extinction correction: *SHELXL97* (Sheldrick, 2008), Fc^*^=kFc[1+0.001xFc^2^λ^3^/sin(2θ)]^-1/4^
Primary atom site location: structure-invariant direct methods	Extinction coefficient: 0.0045 (9)

### Special details

**Table:**

Geometry. All e.s.d.'s (except the e.s.d. in the dihedral angle between two l.s. planes) are estimated using the full covariance matrix. The cell e.s.d.'s are taken into account individually in the estimation of e.s.d.'s in distances, angles and torsion angles; correlations between e.s.d.'s in cell parameters are only used when they are defined by crystal symmetry. An approximate (isotropic) treatment of cell e.s.d.'s is used for estimating e.s.d.'s involving l.s. planes.
Refinement. Refinement of *F*^2^ against ALL reflections. The weighted *R*-factor *wR* and goodness of fit *S* are based on *F*^2^, conventional *R*-factors *R* are based on *F*, with *F* set to zero for negative *F*^2^. The threshold expression of *F*^2^ > σ(*F*^2^) is used only for calculating *R*-factors(gt) *etc*. and is not relevant to the choice of reflections for refinement. *R*-factors based on *F*^2^ are statistically about twice as large as those based on *F*, and *R*- factors based on ALL data will be even larger.

### Fractional atomic coordinates and isotropic or equivalent isotropic displacement parameters (Å^2^)

**Table:**

	*x*	*y*	*z*	*U*_iso_*/*U*_eq_	
O1	0.85799 (14)	0.2548 (4)	0.70663 (5)	0.0333 (7)	
O2	0.71941 (15)	0.6953 (4)	0.72953 (5)	0.0412 (8)	
N1	0.76040 (16)	0.5426 (4)	0.66053 (6)	0.0258 (8)	
N2	0.78287 (15)	0.4506 (4)	0.62766 (6)	0.0242 (8)	
C1	0.83515 (19)	0.2673 (5)	0.63285 (7)	0.0231 (9)	
C2	0.87195 (19)	0.1652 (5)	0.67294 (8)	0.0266 (9)	
C3	0.9241 (2)	−0.0362 (5)	0.67367 (9)	0.0313 (10)	
C4	0.9385 (2)	−0.1299 (6)	0.63794 (9)	0.0353 (10)	
C5	0.9043 (2)	−0.0319 (5)	0.59751 (9)	0.0314 (10)	
C6	0.85416 (19)	0.1681 (5)	0.59467 (8)	0.0269 (9)	
C7	0.8225 (2)	0.2665 (6)	0.55501 (8)	0.0323 (10)	
C8	0.8408 (2)	0.1641 (7)	0.51963 (9)	0.0405 (13)	
C9	0.8902 (2)	−0.0332 (7)	0.52232 (9)	0.0460 (13)	
C10	0.9220 (2)	−0.1313 (6)	0.56069 (10)	0.0417 (11)	
C11	0.67371 (18)	0.8433 (5)	0.61950 (8)	0.0260 (9)	
C12	0.70569 (19)	0.7317 (5)	0.65715 (7)	0.0239 (9)	
C13	0.6843 (2)	0.8137 (5)	0.69426 (8)	0.0294 (10)	
C14	0.6314 (2)	1.0036 (6)	0.69274 (9)	0.0329 (10)	
C15	0.6020 (2)	1.1148 (5)	0.65539 (9)	0.0329 (10)	
C16	0.62311 (19)	1.0343 (5)	0.61810 (8)	0.0290 (9)	
C17	0.5923 (2)	1.1604 (6)	0.57752 (8)	0.0381 (11)	
O5	0.48488 (15)	0.3516 (4)	0.77309 (5)	0.0463 (8)	
O6	0.65278 (14)	0.7820 (4)	0.79689 (5)	0.0320 (7)	
N5	0.65874 (15)	0.5554 (4)	0.87388 (6)	0.0237 (7)	
N6	0.60151 (16)	0.4768 (4)	0.84100 (6)	0.0242 (7)	
C18	0.70644 (18)	0.7387 (5)	0.87009 (7)	0.0221 (9)	
C19	0.70315 (19)	0.8573 (5)	0.83094 (8)	0.0258 (9)	
C20	0.7571 (2)	1.0531 (5)	0.83189 (9)	0.0328 (10)	
C21	0.8074 (2)	1.1348 (5)	0.86792 (9)	0.0334 (10)	
C22	0.81280 (19)	1.0263 (5)	0.90750 (8)	0.0296 (10)	
C23	0.76401 (18)	0.8269 (5)	0.90856 (8)	0.0271 (9)	
C24	0.7715 (2)	0.7172 (6)	0.94742 (8)	0.0318 (10)	
C25	0.8247 (2)	0.8060 (7)	0.98344 (9)	0.0409 (13)	
C26	0.8725 (2)	1.0028 (7)	0.98229 (9)	0.0445 (13)	
C27	0.8668 (2)	1.1131 (6)	0.94502 (10)	0.0398 (11)	
C28	0.55747 (18)	0.1612 (5)	0.88028 (8)	0.0253 (9)	
C29	0.54976 (19)	0.2860 (5)	0.84352 (8)	0.0241 (9)	
C30	0.4884 (2)	0.2180 (5)	0.80699 (8)	0.0290 (9)	
C31	0.4377 (2)	0.0253 (5)	0.80731 (8)	0.0328 (10)	
C32	0.4475 (2)	−0.0984 (5)	0.84354 (8)	0.0313 (10)	
C33	0.5071 (2)	−0.0301 (5)	0.88087 (8)	0.0274 (9)	
C34	0.5164 (2)	−0.1643 (6)	0.92034 (8)	0.0367 (10)	
H1	0.781 (2)	0.481 (5)	0.6851 (5)	0.0489*	
H2	0.70371	0.75370	0.74970	0.0619*	
H3	0.94842	−0.10365	0.69920	0.0376*	
H4	0.97170	−0.26203	0.63954	0.0423*	
H7	0.78964	0.39927	0.55271	0.0386*	
H8	0.81957	0.22875	0.49355	0.0487*	
H9	0.90187	−0.09938	0.49818	0.0552*	
H10	0.95517	−0.26348	0.56240	0.0499*	
H11	0.68711	0.78663	0.59490	0.0311*	
H14	0.61529	1.05717	0.71705	0.0396*	
H15	0.56774	1.24489	0.65496	0.0396*	
H17A	0.55759	1.28918	0.58247	0.0573*	
H17B	0.55310	1.06748	0.55729	0.0573*	
H17C	0.64657	1.20441	0.56707	0.0573*	
H5	0.44769	0.30035	0.75300	0.0694*	
H6	0.598 (2)	0.545 (5)	0.8168 (5)	0.0489*	
H20	0.75780	1.12678	0.80695	0.0393*	
H21	0.83982	1.26671	0.86719	0.0401*	
H24	0.74025	0.58398	0.94863	0.0381*	
H25	0.82845	0.73308	1.00881	0.0489*	
H26	0.90867	1.06058	1.00680	0.0532*	
H27	0.89877	1.24589	0.94449	0.0476*	
H28	0.59739	0.20899	0.90469	0.0303*	
H31	0.39691	−0.02143	0.78307	0.0393*	
H32	0.41389	−0.22936	0.84320	0.0375*	
H34A	0.47613	−0.29120	0.91504	0.0551*	
H34B	0.49865	−0.07553	0.94183	0.0551*	
H34C	0.58041	−0.21161	0.92933	0.0551*	

### Atomic displacement parameters (Å^2^)

**Table:**

	*U*^11^	*U*^22^	*U*^33^	*U*^12^	*U*^13^	*U*^23^
O1	0.0407 (12)	0.0334 (14)	0.0234 (10)	0.0083 (11)	0.0008 (8)	0.0036 (9)
O2	0.0582 (14)	0.0441 (16)	0.0211 (10)	0.0199 (12)	0.0074 (9)	0.0021 (9)
N1	0.0326 (14)	0.0233 (15)	0.0205 (11)	0.0021 (12)	0.0034 (10)	0.0014 (10)
N2	0.0271 (13)	0.0227 (15)	0.0225 (11)	−0.0016 (11)	0.0046 (9)	−0.0026 (10)
C1	0.0230 (14)	0.0216 (17)	0.0234 (13)	−0.0017 (13)	0.0017 (10)	−0.0033 (11)
C2	0.0272 (15)	0.0227 (18)	0.0289 (14)	−0.0002 (13)	0.0036 (11)	0.0022 (12)
C3	0.0305 (16)	0.0249 (19)	0.0352 (15)	0.0003 (14)	−0.0011 (12)	0.0072 (13)
C4	0.0309 (17)	0.0232 (19)	0.0505 (18)	0.0022 (15)	0.0055 (14)	−0.0014 (14)
C5	0.0243 (15)	0.028 (2)	0.0412 (16)	−0.0043 (14)	0.0054 (12)	−0.0087 (14)
C6	0.0255 (15)	0.0253 (19)	0.0278 (14)	−0.0042 (13)	0.0007 (11)	−0.0077 (12)
C7	0.0341 (16)	0.035 (2)	0.0264 (14)	−0.0017 (15)	0.0028 (12)	−0.0045 (13)
C8	0.0388 (18)	0.053 (3)	0.0277 (15)	−0.0015 (18)	0.0021 (13)	−0.0096 (15)
C9	0.0393 (19)	0.058 (3)	0.0396 (18)	−0.0005 (19)	0.0056 (15)	−0.0256 (17)
C10	0.0335 (18)	0.036 (2)	0.055 (2)	0.0026 (16)	0.0075 (15)	−0.0217 (16)
C11	0.0275 (15)	0.0258 (18)	0.0242 (13)	−0.0018 (14)	0.0045 (11)	0.0009 (12)
C12	0.0259 (14)	0.0203 (17)	0.0254 (13)	0.0013 (13)	0.0052 (11)	−0.0005 (11)
C13	0.0329 (16)	0.028 (2)	0.0274 (14)	0.0023 (15)	0.0065 (12)	0.0018 (12)
C14	0.0348 (17)	0.031 (2)	0.0335 (15)	0.0025 (15)	0.0087 (13)	−0.0045 (13)
C15	0.0305 (16)	0.0223 (19)	0.0460 (17)	0.0037 (14)	0.0078 (13)	0.0015 (14)
C16	0.0237 (15)	0.0267 (19)	0.0362 (15)	−0.0006 (14)	0.0052 (12)	0.0091 (13)
C17	0.0390 (18)	0.034 (2)	0.0411 (17)	0.0098 (16)	0.0079 (14)	0.0145 (14)
O5	0.0615 (15)	0.0451 (16)	0.0242 (10)	−0.0219 (13)	−0.0100 (10)	0.0046 (10)
O6	0.0402 (12)	0.0312 (14)	0.0244 (10)	−0.0030 (10)	0.0061 (8)	0.0013 (9)
N5	0.0246 (12)	0.0242 (15)	0.0209 (10)	−0.0002 (11)	0.0018 (9)	−0.0020 (10)
N6	0.0290 (13)	0.0226 (15)	0.0188 (10)	−0.0024 (11)	−0.0002 (10)	−0.0003 (10)
C18	0.0240 (14)	0.0196 (17)	0.0225 (13)	0.0014 (13)	0.0044 (10)	−0.0018 (11)
C19	0.0286 (15)	0.0236 (18)	0.0258 (14)	0.0041 (14)	0.0067 (11)	−0.0002 (12)
C20	0.0340 (17)	0.0259 (19)	0.0403 (16)	−0.0018 (15)	0.0121 (14)	0.0067 (14)
C21	0.0298 (16)	0.0188 (18)	0.0541 (18)	−0.0031 (14)	0.0144 (14)	−0.0020 (14)
C22	0.0276 (16)	0.0257 (19)	0.0364 (15)	0.0008 (14)	0.0085 (12)	−0.0077 (13)
C23	0.0231 (15)	0.0278 (19)	0.0293 (14)	0.0016 (13)	0.0028 (11)	−0.0093 (12)
C24	0.0301 (16)	0.037 (2)	0.0271 (14)	−0.0033 (15)	0.0034 (12)	−0.0060 (13)
C25	0.0365 (18)	0.059 (3)	0.0262 (15)	−0.0017 (18)	0.0040 (12)	−0.0110 (15)
C26	0.0336 (18)	0.063 (3)	0.0361 (17)	−0.0065 (18)	0.0055 (14)	−0.0266 (17)
C27	0.0325 (17)	0.033 (2)	0.0536 (19)	−0.0080 (16)	0.0085 (14)	−0.0188 (16)
C28	0.0251 (15)	0.0258 (18)	0.0237 (13)	0.0019 (13)	0.0022 (11)	0.0017 (12)
C29	0.0250 (14)	0.0210 (17)	0.0254 (13)	0.0002 (13)	0.0030 (11)	−0.0023 (11)
C30	0.0345 (16)	0.0289 (19)	0.0217 (13)	−0.0034 (15)	0.0013 (11)	−0.0002 (12)
C31	0.0326 (17)	0.032 (2)	0.0313 (15)	−0.0076 (15)	0.0006 (12)	−0.0062 (13)
C32	0.0290 (16)	0.0216 (18)	0.0426 (16)	−0.0057 (14)	0.0060 (13)	0.0007 (13)
C33	0.0269 (15)	0.0209 (18)	0.0356 (15)	0.0038 (14)	0.0090 (12)	0.0042 (12)
C34	0.0333 (17)	0.033 (2)	0.0441 (17)	−0.0015 (16)	0.0088 (13)	0.0099 (14)

### Geometric parameters (Å, º)

**Table:**

O1—C2	1.279 (3)	C10—H10	0.9300
O2—C13	1.363 (3)	C11—H11	0.9300
O2—H2	0.8200	C14—H14	0.9300
O5—C30	1.363 (4)	C15—H15	0.9300
O6—C19	1.282 (3)	C17—H17B	0.9600
O5—H5	0.8200	C17—H17A	0.9600
N1—C12	1.385 (4)	C17—H17C	0.9600
N1—N2	1.308 (3)	C18—C19	1.458 (4)
N2—C1	1.336 (4)	C18—C23	1.459 (4)
N1—H1	0.88 (2)	C19—C20	1.418 (4)
N5—C18	1.328 (4)	C20—C21	1.345 (4)
N5—N6	1.305 (3)	C21—C22	1.437 (4)
N6—C29	1.390 (4)	C22—C27	1.413 (4)
N6—H6	0.88 (2)	C22—C23	1.404 (4)
C1—C2	1.446 (4)	C23—C24	1.416 (4)
C1—C6	1.460 (4)	C24—C25	1.377 (4)
C2—C3	1.433 (4)	C25—C26	1.383 (6)
C3—C4	1.351 (4)	C26—C27	1.375 (5)
C4—C5	1.439 (4)	C28—C33	1.372 (4)
C5—C10	1.414 (5)	C28—C29	1.402 (4)
C5—C6	1.406 (4)	C29—C30	1.396 (4)
C6—C7	1.413 (4)	C30—C31	1.381 (4)
C7—C8	1.384 (4)	C31—C32	1.382 (4)
C8—C9	1.387 (6)	C32—C33	1.404 (4)
C9—C10	1.377 (5)	C33—C34	1.505 (4)
C11—C12	1.395 (4)	C20—H20	0.9300
C11—C16	1.366 (4)	C21—H21	0.9300
C12—C13	1.402 (4)	C24—H24	0.9300
C13—C14	1.378 (5)	C25—H25	0.9300
C14—C15	1.381 (4)	C26—H26	0.9300
C15—C16	1.402 (4)	C27—H27	0.9300
C16—C17	1.514 (4)	C28—H28	0.9300
C3—H3	0.9300	C31—H31	0.9300
C4—H4	0.9300	C32—H32	0.9300
C7—H7	0.9300	C34—H34A	0.9600
C8—H8	0.9300	C34—H34B	0.9600
C9—H9	0.9300	C34—H34C	0.9600
			
O1···N1	2.536 (4)	C17···H9^vi^	2.8800
O1···N2	2.843 (4)	C18···H34C^iv^	2.9400
O1···O5^i^	2.622 (3)	C19···H2	2.7300
O2···O6	2.631 (3)	C19···H6	2.42 (3)
O2···N1	2.614 (4)	C20···H32^i^	2.9700
O5···C3^ii^	3.373 (4)	C21···H32^i^	3.1000
O5···O1^ii^	2.622 (3)	C22···H34A^i^	2.7400
O5···N6	2.611 (4)	C23···H34C^iv^	2.9000
O5···C2^ii^	3.344 (4)	C23···H34A^i^	3.0600
O6···N6	2.546 (4)	C24···H34C^iv^	2.7500
O6···N5	2.848 (4)	C25···H24^vii^	3.0800
O6···O2	2.631 (3)	C26···H24^vii^	3.0900
O1···H1	1.82 (3)	C27···H34A^i^	2.8100
O1···H5^i^	1.8100	C31···H21^viii^	2.9300
O2···H1	2.26 (3)	C32···H21^viii^	2.7500
O5···H3^ii^	2.8100	C33···H21^viii^	2.8700
O5···H6	2.27 (3)	C34···H26^ix^	2.9200
O6···H6	1.82 (3)	H1···O2	2.26 (3)
O6···H2	1.8500	H1···C2	2.40 (3)
N1···C15^iii^	3.446 (5)	H1···O1	1.82 (3)
N1···O1	2.536 (4)	H2···H14	2.3700
N1···O2	2.614 (4)	H2···O6	1.8500
N2···O1	2.843 (4)	H2···C19	2.7300
N2···C4^iv^	3.372 (5)	H3···O5^i^	2.8100
N2···C16^iii^	3.398 (5)	H3···H5^i^	2.5400
N2···C17^iii^	3.402 (5)	H4···C15^i^	2.8300
N5···O6	2.848 (4)	H4···H10	2.4800
N5···C33^iv^	3.378 (5)	H4···C14^i^	2.9800
N5···C34^iv^	3.279 (5)	H4···C16^i^	2.9500
N5···C21^iii^	3.371 (5)	H5···C2^ii^	2.6300
N6···O5	2.611 (4)	H5···C3^ii^	2.9100
N6···O6	2.546 (4)	H5···H3^ii^	2.5400
N6···C32^iv^	3.422 (5)	H5···H31	2.3700
N2···H7	2.4900	H5···O1^ii^	1.8100
N2···H11	2.5700	H6···C19	2.42 (3)
N2···H17C^iii^	2.9100	H6···O5	2.27 (3)
N5···H34C^iv^	2.7200	H6···O6	1.82 (3)
N5···H24	2.4900	H7···N2	2.4900
N5···H28	2.5600	H7···H17C^iii^	2.5200
C1···C11^iii^	3.445 (5)	H9···C17^x^	2.8800
C1···C16^iii^	3.334 (5)	H9···H17C^x^	2.4100
C2···C12^iii^	3.533 (5)	H10···H4	2.4800
C2···O5^i^	3.344 (4)	H10···C17^i^	3.1000
C3···O5^i^	3.373 (4)	H10···H17B^i^	2.3500
C3···C12^iii^	3.413 (5)	H11···N2	2.5700
C4···N2^iii^	3.372 (5)	H14···H2	2.3700
C6···C11^iii^	3.504 (5)	H15···H17A	2.3500
C11···C6^iv^	3.504 (5)	H15···C2^viii^	3.0700
C11···C1^iv^	3.445 (5)	H15···C3^viii^	2.8900
C12···C3^iv^	3.413 (5)	H15···C4^viii^	2.9700
C12···C2^iv^	3.533 (5)	H17A···H15	2.3500
C15···N1^iv^	3.446 (5)	H17A···C5^viii^	2.8000
C16···C1^iv^	3.334 (5)	H17A···C6^viii^	3.0800
C16···N2^iv^	3.398 (5)	H17A···C10^viii^	2.8500
C17···N2^iv^	3.402 (5)	H17B···H10^ii^	2.3500
C18···C33^iv^	3.302 (5)	H17C···N2^iv^	2.9100
C18···C28^iv^	3.411 (5)	H17C···C6^iv^	2.9800
C18···C34^iv^	3.540 (5)	H17C···C7^iv^	2.6900
C19···C28^iv^	3.445 (5)	H17C···H7^iv^	2.5200
C19···C29^iv^	3.500 (5)	H17C···H9^vi^	2.4100
C20···C29^iv^	3.419 (5)	H21···H27	2.4900
C21···N5^iv^	3.371 (5)	H21···C31^v^	2.9300
C23···C28^iv^	3.581 (5)	H21···C32^v^	2.7500
C28···C19^iii^	3.445 (5)	H21···C33^v^	2.8700
C28···C18^iii^	3.411 (5)	H24···N5	2.4900
C28···C23^iii^	3.581 (5)	H24···H34C^iv^	2.5900
C29···C19^iii^	3.500 (5)	H24···C25^xi^	3.0800
C29···C20^iii^	3.419 (5)	H24···C26^xi^	3.0900
C32···N6^iii^	3.422 (5)	H26···C34^xii^	2.9200
C33···C18^iii^	3.302 (5)	H26···H34C^xii^	2.4800
C33···N5^iii^	3.378 (5)	H27···H21	2.4900
C34···N5^iii^	3.279 (5)	H27···H34B^v^	2.4800
C34···C18^iii^	3.540 (5)	H28···N5	2.5600
C2···H15^v^	3.0700	H31···H5	2.3700
C2···H1	2.40 (3)	H32···H34A	2.3600
C2···H5^i^	2.6300	H32···C20^ii^	2.9700
C3···H15^v^	2.8900	H32···C21^ii^	3.1000
C3···H5^i^	2.9100	H34A···H32	2.3600
C4···H15^v^	2.9700	H34A···C22^ii^	2.7400
C5···H17A^v^	2.8000	H34A···C23^ii^	3.0600
C6···H17A^v^	3.0800	H34A···C27^ii^	2.8100
C6···H17C^iii^	2.9800	H34B···H27^viii^	2.4800
C7···H17C^iii^	2.6900	H34C···N5^iii^	2.7200
C10···H17A^v^	2.8500	H34C···C18^iii^	2.9400
C14···H4^ii^	2.9800	H34C···C23^iii^	2.9000
C15···H4^ii^	2.8300	H34C···C24^iii^	2.7500
C16···H4^ii^	2.9500	H34C···H24^iii^	2.5900
C17···H10^ii^	3.1000	H34C···H26^ix^	2.4800
			
C13—O2—H2	109.00	H17B—C17—H17C	109.00
C30—O5—H5	109.00	C16—C17—H17A	109.00
N2—N1—C12	121.5 (2)	C16—C17—H17B	109.00
N1—N2—C1	118.8 (2)	H17A—C17—H17B	109.00
N2—N1—H1	118.1 (18)	C16—C17—H17C	109.00
C12—N1—H1	120.4 (18)	N5—C18—C23	116.4 (2)
N6—N5—C18	119.0 (2)	C19—C18—C23	118.9 (3)
N5—N6—C29	121.0 (2)	N5—C18—C19	124.7 (2)
N5—N6—H6	118.3 (18)	O6—C19—C20	122.1 (3)
C29—N6—H6	120.6 (18)	O6—C19—C18	119.6 (3)
N2—C1—C6	115.9 (2)	C18—C19—C20	118.3 (2)
C2—C1—C6	119.8 (3)	C19—C20—C21	121.7 (3)
N2—C1—C2	124.4 (2)	C20—C21—C22	122.5 (3)
O1—C2—C3	121.6 (2)	C23—C22—C27	119.5 (3)
C1—C2—C3	118.3 (2)	C21—C22—C23	118.7 (2)
O1—C2—C1	120.1 (3)	C21—C22—C27	121.8 (3)
C2—C3—C4	121.2 (3)	C18—C23—C22	119.8 (2)
C3—C4—C5	122.2 (3)	C18—C23—C24	121.6 (3)
C4—C5—C6	119.5 (3)	C22—C23—C24	118.6 (3)
C4—C5—C10	121.0 (3)	C23—C24—C25	120.5 (3)
C6—C5—C10	119.5 (3)	C24—C25—C26	120.7 (3)
C1—C6—C7	121.7 (3)	C25—C26—C27	120.2 (3)
C1—C6—C5	119.0 (2)	C22—C27—C26	120.5 (3)
C5—C6—C7	119.3 (3)	C29—C28—C33	121.2 (2)
C6—C7—C8	119.6 (3)	N6—C29—C28	123.3 (2)
C7—C8—C9	121.3 (3)	N6—C29—C30	116.9 (2)
C8—C9—C10	120.1 (3)	C28—C29—C30	119.8 (3)
C5—C10—C9	120.2 (3)	O5—C30—C29	115.4 (3)
C12—C11—C16	121.3 (2)	O5—C30—C31	125.2 (2)
N1—C12—C11	123.5 (2)	C29—C30—C31	119.4 (3)
N1—C12—C13	116.7 (2)	C30—C31—C32	120.2 (3)
C11—C12—C13	119.8 (3)	C31—C32—C33	121.3 (3)
O2—C13—C14	125.3 (2)	C28—C33—C32	118.2 (2)
C12—C13—C14	119.1 (3)	C28—C33—C34	120.9 (2)
O2—C13—C12	115.6 (3)	C32—C33—C34	121.0 (3)
C13—C14—C15	120.5 (3)	C19—C20—H20	119.00
C14—C15—C16	120.9 (3)	C21—C20—H20	119.00
C15—C16—C17	120.4 (3)	C20—C21—H21	119.00
C11—C16—C15	118.5 (3)	C22—C21—H21	119.00
C11—C16—C17	121.1 (2)	C23—C24—H24	120.00
C4—C3—H3	119.00	C25—C24—H24	120.00
C2—C3—H3	119.00	C24—C25—H25	120.00
C5—C4—H4	119.00	C26—C25—H25	120.00
C3—C4—H4	119.00	C25—C26—H26	120.00
C6—C7—H7	120.00	C27—C26—H26	120.00
C8—C7—H7	120.00	C22—C27—H27	120.00
C7—C8—H8	119.00	C26—C27—H27	120.00
C9—C8—H8	119.00	C29—C28—H28	119.00
C8—C9—H9	120.00	C33—C28—H28	119.00
C10—C9—H9	120.00	C30—C31—H31	120.00
C5—C10—H10	120.00	C32—C31—H31	120.00
C9—C10—H10	120.00	C31—C32—H32	119.00
C16—C11—H11	119.00	C33—C32—H32	119.00
C12—C11—H11	119.00	C33—C34—H34A	109.00
C13—C14—H14	120.00	C33—C34—H34B	109.00
C15—C14—H14	120.00	C33—C34—H34C	109.00
C14—C15—H15	120.00	H34A—C34—H34B	109.00
C16—C15—H15	120.00	H34A—C34—H34C	109.00
H17A—C17—H17C	109.00	H34B—C34—H34C	109.00
			
C12—N1—N2—C1	−179.4 (3)	O2—C13—C14—C15	177.8 (3)
N2—N1—C12—C11	−2.7 (4)	C12—C13—C14—C15	−1.7 (5)
N2—N1—C12—C13	178.8 (3)	C13—C14—C15—C16	1.7 (5)
N1—N2—C1—C2	−2.1 (4)	C14—C15—C16—C17	−178.7 (3)
N1—N2—C1—C6	177.4 (2)	C14—C15—C16—C11	−0.2 (4)
N6—N5—C18—C19	−2.8 (4)	N5—C18—C19—O6	−1.4 (4)
N6—N5—C18—C23	175.7 (2)	N5—C18—C19—C20	179.1 (3)
C18—N5—N6—C29	−179.7 (3)	C23—C18—C19—O6	−179.9 (3)
N5—N6—C29—C28	−2.5 (4)	C23—C18—C19—C20	0.6 (4)
N5—N6—C29—C30	178.2 (3)	N5—C18—C23—C22	−176.8 (3)
N2—C1—C6—C5	−176.5 (3)	N5—C18—C23—C24	2.6 (4)
N2—C1—C6—C7	2.7 (4)	C19—C18—C23—C22	1.8 (4)
N2—C1—C2—O1	−2.6 (4)	C19—C18—C23—C24	−178.8 (3)
N2—C1—C2—C3	177.7 (3)	O6—C19—C20—C21	177.9 (3)
C6—C1—C2—O1	177.9 (3)	C18—C19—C20—C21	−2.7 (4)
C6—C1—C2—C3	−1.9 (4)	C19—C20—C21—C22	2.3 (5)
C2—C1—C6—C5	3.1 (4)	C20—C21—C22—C23	0.3 (4)
C2—C1—C6—C7	−177.7 (3)	C20—C21—C22—C27	179.3 (3)
C1—C2—C3—C4	−0.3 (4)	C21—C22—C23—C18	−2.3 (4)
O1—C2—C3—C4	180.0 (3)	C21—C22—C23—C24	178.3 (3)
C2—C3—C4—C5	1.2 (5)	C27—C22—C23—C18	178.7 (3)
C3—C4—C5—C6	0.0 (5)	C27—C22—C23—C24	−0.8 (4)
C3—C4—C5—C10	178.7 (3)	C21—C22—C27—C26	−178.4 (3)
C4—C5—C6—C7	178.6 (3)	C23—C22—C27—C26	0.6 (5)
C4—C5—C6—C1	−2.2 (4)	C18—C23—C24—C25	−178.7 (3)
C10—C5—C6—C7	−0.1 (4)	C22—C23—C24—C25	0.8 (4)
C4—C5—C10—C9	−178.8 (3)	C23—C24—C25—C26	−0.6 (5)
C10—C5—C6—C1	179.1 (3)	C24—C25—C26—C27	0.5 (5)
C6—C5—C10—C9	−0.1 (5)	C25—C26—C27—C22	−0.5 (5)
C5—C6—C7—C8	0.4 (4)	C33—C28—C29—N6	−177.9 (3)
C1—C6—C7—C8	−178.8 (3)	C33—C28—C29—C30	1.4 (4)
C6—C7—C8—C9	−0.4 (5)	C29—C28—C33—C32	0.1 (4)
C7—C8—C9—C10	0.1 (5)	C29—C28—C33—C34	179.4 (3)
C8—C9—C10—C5	0.2 (5)	N6—C29—C30—O5	−1.6 (4)
C16—C11—C12—N1	−177.3 (3)	N6—C29—C30—C31	177.7 (3)
C12—C11—C16—C17	177.2 (3)	C28—C29—C30—O5	179.1 (3)
C12—C11—C16—C15	−1.3 (4)	C28—C29—C30—C31	−1.6 (4)
C16—C11—C12—C13	1.2 (4)	O5—C30—C31—C32	179.4 (3)
N1—C12—C13—C14	178.9 (3)	C29—C30—C31—C32	0.2 (4)
N1—C12—C13—O2	−0.6 (4)	C30—C31—C32—C33	1.4 (5)
C11—C12—C13—C14	0.3 (4)	C31—C32—C33—C28	−1.5 (4)
C11—C12—C13—O2	−179.3 (3)	C31—C32—C33—C34	179.2 (3)

Symmetry codes: (i) *x*+1/2, −*y*+1/2, *z*; (ii) *x*−1/2, −*y*+1/2, *z*; (iii) *x*, *y*−1, *z*; (iv) *x*, *y*+1, *z*; (v) *x*+1/2, −*y*+3/2, *z*; (vi) −*x*+3/2, *y*+3/2, −*z*+1; (vii) −*x*+3/2, *y*+1/2, −*z*+2; (viii) *x*−1/2, −*y*+3/2, *z*; (ix) −*x*+3/2, *y*−3/2, −*z*+2; (x) −*x*+3/2, *y*−3/2, −*z*+1; (xi) −*x*+3/2, *y*−1/2, −*z*+2; (xii) −*x*+3/2, *y*+3/2, −*z*+2.

### Hydrogen-bond geometry (Å, º)

**Table:**

*D*—H···*A*	*D*—H	H···*A*	*D*···*A*	*D*—H···*A*
N1—H1···O1	0.88 (2)	1.82 (3)	2.536 (4)	138 (2)
O2—H2···O6	0.82	1.85	2.631 (3)	159
O5—H5···O1^ii^	0.82	1.81	2.622 (3)	168
N6—H6···O6	0.88 (2)	1.82 (3)	2.546 (4)	138 (2)

Symmetry code: (ii) *x*−1/2, −*y*+1/2, *z*.
